# Filamentous phage associated with recent pandemic strains of Vibrio parahaemolyticus.

**DOI:** 10.3201/eid0703.010325

**Published:** 2001

**Authors:** T Iida, A Hattori, K Tagomori, H Nasu, R Naim, T Honda

**Affiliations:** Osaka University, Suita, Osaka, Japan. iida@biken.osaka-u.ac.jp

## Abstract

A group of pandemic strains of Vibrio parahaemolyticus has recently appeared in Asia and North America. We demonstrate that a filamentous phage is specifically associated with the pandemic V. parahaemolyticus strains. An open reading frame unique to the phage is a useful genetic marker to identify these strains.


### Filamentous Phage Associated with Recent Pandemic Strains of *Vibrio parahaemolyticus*

**Tetsuya Iida, Akiko Hattori, Kenichi Tagomori, Hatsumi Nasu, Rochman Naim, and Takeshi Honda**
Osaka University, Suita, Osaka, Japan

A group of pandemic strains of *Vibrio parahaemolyticus* has recently appeared in Asia and North America. We demonstrate that a filamentous phage is specifically associated with the pandemic *V. parahaemolyticus* strains. An open reading frame unique to the phage is a useful genetic marker to identify these strains.

*Vibrio parahaemolyticus*, a halophilic gram-negative rod, causes seafood-borne gastroenteritis in humans. Infections caused by this organism have been associated with diverse serovars: 13 O serotypes and 75 K serotypes have been identified. Recent studies, however, have revealed the emergence and pandemic spread of a single serovar, O3:K6 (1-6). Strains belonging to the O3:K6 serovar abruptly appeared in India in 1996 and have since been isolated in Southeast Asian countries, from travelers at quarantine stations in Japan, and from foodborne outbreaks in the United States (1-5). This serovar accounts for more than half of the *V. parahaemolyticus* isolates from diarrheal patients in Japan (6). Such widespread occurrence of a single serovar of *V. parahaemolyticus* had not previously been reported. Since 1998, *V. parahaemolyticus* strains belonging to other two serovars, O4:K68 and O1:K untypeable (KUT), have also been isolated with increasing frequency from diarrheal patients (2,6,7). The genetic background of the O4:K68 and O1:KUT isolates is almost indistinguishable from that of the recent O3:K6 strains, suggesting a common origin (2,7).

In a previous study, we reported on a filamentous phage that is specifically associated with the recent O3:K6 serovar strains of *V. parahaemolyticus* (8). This phage, f237, has several genes in common with and a similar genomic structure to another filamentous phage, CTX (9), which is known to carry the genes for cholera enterotoxin (*ctxAB*), the most important virulence factor of *V. cholerae*. Instead of *ctxAB*, f237 possesses a unique open reading frame, ORF8, which has no homology with other sequences in DNA databases (8). In this study, we examined the distribution of f237 in recent clinical isolates of *V. parahaemolyticus*.

### The Study

We studied 96 strains of *V. parahaemolyticus* isolated from diarrheal patients during January to May 1999 at the Kansai International Airport quarantine station, Osaka, Japan. Detection of f237 was by colony hybridization using a digoxigenin-labeled DNA probe targeted for ORF8 (8) and prepared as described (8). In brief, after amplification of a partial DNA sequence of ORF8 (746 bp in size) by polymerase chain reaction (PCR) with the genomic DNA of *V. parahaemolyticus* KXV237 strain (RIMD2210633) (8) as a template, the amplified DNA was labeled with digoxigenin using a PCR DIG labeling kit (Boehringer GmbH, Mannheim, Germany). PCR primers for ORF8 were 8S (5'-GTTCGCATA-CAGTTGAGG-3') and 8A (5'-AAGTACAGCAGGAGTGAG-3'). The conditions for PCR were as follows: After heating at 94°C for 3 minutes, a cycle of 94°C for 30 seconds, 57°C for 30 seconds, and 72°C for 1 minute was repeated 30 times, followed by end extension step at 72°C for 5 minutes. PCR was done on a Perkin-Elmer (Foster City, CA) thermal cycler type 9700. Pulsed-field gel electrophoresis (PFGE) and Southern hybridization were performed as described (10). For PFGE, the *V. parahaemolyticus* genomic DNA in agarose blocks was digested with restriction enzymes *Not*I or *Sfi*I. A contour-clamped homogeneous electric field method was used for PFGE on a CHEF Mapper System (Bio-Rad Laboratories, Richmond, CA). The conditions for PFGE were 1% agarose gel in 0.5x Tris-borate-EDTA buffer at 6 V $cm^{-1}$, with pulse times increasing linearly from 1 to 60 seconds within 24 hours.

Of the 96 strains examined, 53 tested positive for ORF8, suggesting the presence of phage f237 (Table). In addition to O3:K6, the ORF8-positive strains were found in strains with serotypes O4:K68 and O1:KUT. None of the strains with other serovars showed evidence of ORF8. When the genomic DNA of the strains was digested with *Not*I or *Sfi*I endonuclease, PFGE showed very similar restriction patterns for the bacterial genomes of the ORF8-positive strains, irrespective

Table. Distribution of ORF8 in clinical isolates of *Vibrio parahaemolyticus*

| Serovar | ORF8[a] | PFGE genotype[b] | No. of strains |
|---|---|---|---|
| O3:K6 | + | A | 34 |
| | - | A | 1 |
| O4:K68 | + | A | 11 |
| | - | - | 0 |
| O1:KUT | + | A | 8 |
| | - | A | 1 |
| | - | B | 8 |
| Others | + | - | 0 |
| | - | B | 33 |
| Total | | | 96 |

[a]Possession of ORF8: (+) denotes presence of ORF8; (-) denotes absence of ORF8.
[b]Pulsed-field gel electrophoresis (PFGE) pattern of the genomic DNA of strains; A, genotype closely related to recent O3:K6 strains (7); B, genotype distinct from that of recent O3:K6 strains.

Address for correspondence: Tetsuya Iida, Department of Bacterial Infections, Research Institute for Microbial Diseases, Osaka University, 3-1 Yamadaoka, Suita, Osaka 565-0871, Japan; fax: 81-6-6879-8277; e-mail: iida@biken.osaka-u.ac.jp of their serovars (data not shown). Southern hybridization with a probe for ORF8 after PFGE demonstrated that, in all the ORF8-positive strains, the largest *Not*I fragment (size approximately 1,080 kb) reacted with the probe, suggesting that f237 integrated into the bacterial chromosome.

### Conclusions

Our study shows that the filamentous phage f237 is associated not only with O3:K6 serovar but also with other recently emerging serovars of *V. parahaemolyticus*. In the 55 strains showing PFGE genotypes closely related to those of the recent O3:K6 strains, >96% was positive for the ORF8 (Table). Such high prevalence of the phage f237 in the *V. parahaemolyticus* strains showing pandemic spread suggests that the phage might confer a so-far-unknown phenotype to the bacterium. The phenotype might, in turn, protect the organism against selective pressure in a certain environment before it infects humans. Whether phage f237 has played a part in the recently increasing pandemic potency of strains of *V. parahaemolyticus* is a subject for further study.

The virulence of *V. parahaemolyticus* extends beyond acute gastroenteritis: it can also cause wound infections and septicemia (5). Physicians everywhere need to be alert to the possibility of infection from these recently emergent strains. To distinguish the new and increasingly common *V. parahaemolyticus* strains from classic ones, ORF8 is a useful genetic marker.

### Acknowledgments

The authors thank the staff at the Kansai International Airport quarantine station for providing the *V. parahaemolyticus* strains used in this study.

This work was supported by a grant-in-aid for scientific research from the Ministry of Education, Science, Sports, and Culture of Japan and the "Research for the Future" Program of the Japan Society for the Promotion of Sciences (JSPS-RFTF 97L00704).

Dr. Iida is an associate professor in the Department of Bacterial Infections, Research Institute for Microbial Diseases, Osaka University, Japan. His research interests include the mechanism and evolution of bacterial pathogenesis, especially of enteropathogens such as vibrios and *Escherichia coli*.

### References

1. Okuda J, Ishibashi M, Hayakawa E, Nishino T, Takeda Y, Mukhopadhyay AK, et al. Emergence of a unique O3:K6 clone of *Vibrio parahaemolyticus* in Calcutta, India, and isolation of strains from the same clonal group from Southeast Asian travelers arriving in Japan. J Clin Microbiol 1997;35:3150-5.
2. Matsumoto C, Okuda J, Ishibashi M, Iwanaga M, Garg P, Rammamurthy T, et al. Pandemic spread of an O3:K6 clone of *Vibrio parahaemolyticus* and emergence of related strains evidenced by arbitrarily primed PCR and *toxRS* sequence analyses. J Clin Microbiol 2000;38:578–85.
3. Centers for Disease Control and Prevention. Outbreak of *Vibrio parahaemolyticus* infections associated with eating raw oysters—Pacific Northwest, 1997. MMWR Morb Mortal Wkly Rep 1998;47:457-62.
4. Centers for Disease Control and Prevention. Outbreak of *Vibrio parahaemolyticus* infection associated with eating raw oysters and clams harvested from Long Island Sound—Connecticut, New Jersey, and New York, 1998. MMWR Morb Mortal Wkly Rep 1999;48:48-51.
5. Daniels NA, MacKinnon L, Bishop R, Altekruse S, Ray B, Hammond RM, et al. *Vibrio parahaemolyticus* infections in the United States, 1973–1998. J Infect Dis 2000;181:1661–6.
6. World Health Organization. *Vibrio parahaemolyticus*, Japan, 1996-1998. Wkly Epidemiol Rec 1999;74:361-3.
7. Chowdhury NR, Chakraborty S, Eampokalap B, Chaicumpa W, Chongsa-Nguan M, Moolasart P, et al. Clonal dissemination of *Vibrio parahaemolyticus* displaying similar DNA fingerprint but belonging to two different serovars (O3:K6 and O4:K68) in Thailand and India. Epidemiol Infect 2000;125:17-25.
8. Nasu H, Iida T, Sugahara T, Yamaichi Y, Park K-S, Yokoyama K, et al. A filamentous phage associated with recent pandemic *Vibrio parahaemolyticus* O3:K6 strains. J Clin Microbiol 2000;38:2156–61.
9. Waldor MK, Mekalanos JJ. Lysogenic conversion by a filamentous phage encoding cholera toxin. Science 1996;272:1910–4.
10. Yamaichi Y, Iida T, Park K-S, Yamamoto K, Honda T. Physical and genetic map of the genome of *Vibrio parahaemolyticus*: presence of two chromosomes in *Vibrio* species. Mol Microbiol 1999;31:1513-21.
